# Characterization of Nuclear Progesterone Receptor Isoforms in the Term Equine Placenta

**DOI:** 10.3389/fvets.2021.660177

**Published:** 2021-04-01

**Authors:** Ahmed M. Nagy, Swanand R. Sathe, Attia H. Atta, Abdel Mohsen M. Hammam, Walter H. Hsu

**Affiliations:** ^1^Department of Animal Reproduction and Artificial Insemination, Veterinary Research Division, National Research Center, Cairo, Egypt; ^2^Department of Veterinary Clinical Sciences, College of Veterinary Medicine, Iowa State University, Ames, IA, United States; ^3^Department of Pharmacology, Faculty of Veterinary Medicine, Cairo University, Cairo, Egypt; ^4^Department of Biomedical Sciences, College of Veterinary Medicine, Iowa State University, Ames, IA, United States

**Keywords:** placenta, equine, progesterone, receptors, PRA, PRB

## Abstract

In equine parturition, the role of progestins along with the nuclear progesterone receptor (nPR) signaling pathway in the placenta is not completely clarified. The progestins play an integral role in maintaining myometrial quiescence during the late stage of pregnancy via acting on nPR isoforms (PRA and PRB; PRB is more active than PRA). The current study aimed to determine the PRA and PRB expressions in the term equine placenta at the gene and protein levels. Six term equine placentas were used in this study. Reverse transcription polymerase chain reaction (RT-PCR) was used to quantify the mRNA expression for PRA and PRB. The protein expression was detected using the Western Blot technique. The results revealed that the mRNA and protein expressions for PRA were significantly higher (*P* < 0.0001) in the term equine placental tissue compared to the mRNA and protein expressions of PRB. These results demonstrated that nPRs are detectable in the term placenta of mares and PRA is the dominant isoform expressed. The present findings raised the possibility that the PRA plays an important role in the parturition process and expulsion of the placenta in mares.

## Introduction

The placenta is a crucial organ for a successful pregnancy establishment. It provides oxygen and nutrients to the conceptus (1, 2), as well as acts as an endocrine organ that provides steroid hormones including estradiol (E2) and progesterone (P4) (3, 4). P4 is the core component of pregnancy maintenance in mammals. The P4 performs its action non-genomically through binding to membrane receptors and genomically via binding to nuclear receptors (nPRs) (5–7). There are two isoforms of nPRs; PRA and PRB which are encoded by the same gene, but regulated by different promoters (8–10). PRA is the less active or inactive form of P4 receptors and shorter in amino acid sequence than PRB, the active form of the receptors (11–13).

In humans and other primates, P4 is crucial for maintaining the pregnancy (14, 15). However, the gestational physiology of mares is intricate. In mares, pregnancy is maintained even with undetectable plasma P4 concentrations (<0.5 ng/mL) starting in mid-pregnancy. Recently, it was reported that other pregnanes, mainly 5 α-dihydroprogesterone (5α-DHP) and 3α-DHP, maintain the pregnancy until term (16–18). The potency of DHP is equivalent to P4 on equine PR (18). Moreover, the parturition process in equine is accompanied by a drop of the DHP levels (progestin withdrawal) a few days before foaling (19). Very recent studies suggested that the infectious placentitis in equine is accompanied by progestin withdrawal, a decrease of the PR mRNA expression and a small degree of decline of the PRB protein in the myometrium which parallels with an increase of the inflammatory cytokines triggering the myometrial activation (20, 21).

The myometrium is the key maternal tissue for parturition (22–25). There is an interesting question as to whether the equine placenta is involved in the feto-maternal signaling pathway during parturition. For decades, it was well-known that P4 exerts its effect on the uterus and indirectly regulates placental function, whereas the existence of nPRs in the placenta is not completely understood (26–29). However, studies in humans suggested that the placenta is one of the target tissues for the P4 effect (3, 30, 31). The changes in the expression of nPR isoforms in the human placenta and its role in the feto-maternal signaling pathway for initiation of parturition have been revealed (32, 33). However, little is known about the changes of nPR isoforms in the equine placenta during parturition. To our best knowledge, this is the first study that determined both nPRs; mRNA and protein expressions of PRA and PRB in the equine placenta at the normal term labor.

We hypothesized that both nPR isoforms (PRA and PRB) exist in the term equine placenta. Thus, the present study aimed to determine the PRA and PRB expressions in the equine term placenta at the gene and protein levels.

## Materials and Methods

### Placental Tissue Processing

Placentas from Quarter Horse mares were collected immediately after foaling (*n* = 6) in the Large Animal Hospital, College of Veterinary Medicine, Iowa State University. This study was approved by the Institutional Animal Care and Use Committee of Iowa State University. The age of the mares was 7–10 years. The foaling processes were without complications and the gestation lengths were ranged between 332 and 358 days. The placentas were weighed and examined for completeness and abnormalities shortly after foaling. All placentas were normal. The tissue samples were taken from the chorioallantoic region. Immediately, the samples were dissected to remove the connective tissue, washed with chilled phosphate-buffered saline (PBS), immersed in liquid nitrogen and stored at −80°C for further studies.

### RNA Isolation and cDNA Synthesis

Total RNA was isolated using the Absolutely RNA Miniprep Kit RNA (Agilent Technologies, Santa Clara, CA, USA) according to the manufacturer's instructions. The concentration and purity of the isolated RNA were determined by the Nanodrop 2000 spectrophotometer (Thermo Scientific, St Peters, MO, USA) by measuring the wavelengths at 260 and 280 nm, respectively. Total RNA (500 ng) was reverse-transcribed with random primers using the high capacity RNA to cDNA Kit (Applied Biosystems, Fermont, CA, USA) following the manufacturer's instructions.

### Quantitative RT-PCR

The PCR reaction was performed using the Quant Studio™ 3 System (Applied Biosystems, Fermont, CA, USA). All samples were performed in triplicates using Power ™ SYBR Green Master Mix (Applied Biosystems, Cat# 43-676-59) along with specific primers (Table 1) designed using the Invitrogen primer design tool according to published sequences in the NCBI database and synthesized at Iowa State University's DNA Facility. Two sets of primers were designed for nuclear progesterone receptors. One is attached to the PR common sequence and detects both PRA and PRB. The other one is specific for the N-terminus of the PRB and detects only PRB. Double distilled water without cDNA template was used as a negative control in the PCR reaction. The amount of mRNA encoding PRA was calculated by subtracting the relative abundance of PRB mRNA from that of total PR mRNA. PCR efficiency was estimated using LinRegPCR (version 11.0). The relative mRNA abundance for the gene of interest was expressed as arbitrary units compared to the β-actin housekeeping gene (ACTB) mRNA using the ΔΔCT method as described (34).

**Table 1:** Sequences of the qPCR primers according to their accession numbers.

**Gene**	**Primer**	**Accession no**.
PRAB	F: TACCTGAGGCCGGATTCAGA R: GCTCCCACAGGTAAGGACAC	KJ197859
PRB	F: GCTGGACAGTGTCTTGGACA R: GCAGATCGGGGATCTTCAGG	KJ197859
ACTB	F: TCACCAACTGGGACGACATG R: AGTCCATCACGATGCCAGTG	AF035774

*PR, Progesterone receptor; ACTB, β-actin*.

### Protein Isolation and Quantification

Frozen placental samples (~100 mg) were dissected and homogenized in the radioimmunoprecipitation assay buffer (RIPA,150 mM NaCl, 1%Triton X-100, 0.5% sodium deoxycholate, 0.1% SDS, 50 mM Tris, pH 8.0) with Halt™protease inhibitor cocktail (Thermo Scientific Corporation, St Peters, MO, USA) using the Tissumizer (Tekmar, Cincinnati, OH, USA). The protein concentration of each lysate was determined using the BCA assay (Pierce, Thermo scientific corporation, Cleveland, OH, USA).

### Western Blot Analysis

The extracted proteins of 40 μg were diluted in 2x gel loading buffer (40% glycerol, 1 M Tris-HCl, pH 6.8, 2.5% β-mercaptoethanol, 8% sodium, dodecyl sulfate (SDS), and 0.01% bromophenol blue). The mixture was heated for 5–10 min at 100°C for complete denaturation. The denatured proteins were separated by polyacrylamide gel electrophoresis with SDS (10% SDS–PAGE) using Mini-PROTEAN System® (Bio-Rad, Hercules, CA, USA). T47D whole cell lysate (Santa Cruz Biotechnology, Santa Cruz, CA, USA, Cat# SC-2293) was used as the positive control for PRA and PRB in Western Blot analysis. The separated proteins were transferred to nitrocellulose membranes (Pierce, Thermo scientific corporation, Cleveland, OH, USA) using the Criterion™ System (Bio-Rad, Hercules, CA, USA). Subsequently, the membranes were blocked with 5% dry skimmed milk in Tris-buffered saline and Tween 20 (TBST) buffer (100 mM Tris–HCl, 0.9% NaCl, and 0.05% Tween 20) for 1 h at room temperature. The membranes were incubated overnight at 4°C with hPRa6 monoclonal antibody which recognizes both PRA and PRB (Invitrogen, Thermo Scientific Corporation, Cat# MA5-12653) and anti β-actin antibody (Santa Cruz Biotechnology, Cat# Sc-81178) as a loading control at 1:200 dilution. Membranes were washed 5 times for 5 min each with TBST buffer and incubated with the secondary antibody (Alex fluor®680 goat anti-mouse antibody) at 1:10,000 dilution (Invitrogen, Thermo Scientific Corporation, Cat# A-21058) for 1 h at room temperature. Then, it was washed again before being applied to the imaging system. The LI-COR Odyssey imaging system was used to scan the membrane and imaging to detect the immunofluorescent bands. Band intensity was measured by Image J analysis software.

### Data Analysis

The data are presented as the mean ± SEM and were statistically analyzed using Student's *t*-test after checking for normality. The *P* < 0.05 was treated as statistically significant. The qPCR data were normalized using β-actin as a housekeeping gene and analyzed using the ΔΔCT method to obtain the relative abundance for mRNA encoding PRA and PRB (34). The amount of mRNA encoding PRA was calculated by subtracting the relative abundance of PRB mRNA from that of total PR mRNA. The relative abundance of PRA and PRB was obtained by dividing their values by β-actin values. The band intensity of the PR proteins and calibrator was identified using Image J software Graph Pad Prism 5.0 software package (GraphPad Software, Inc., San Diego, CA, USA) was used for performing all the calculations (35).

## Results

### PRA and PRB mRNA in Equine Placenta

**Figure 1:**
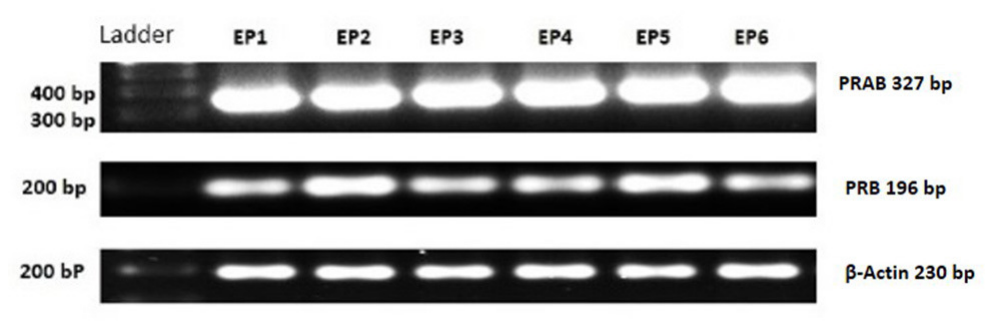
PCR products for PRAB, PRB, and housekeeping gene (β-Actin) of equine placental tissue samples (EP) 1–6. Bands appear at the expected size (PRAB 327 bp, PRB 196 bp and β-Actin 230 bp).

The mRNA encoding PRA and PRB have been confirmed according to the amplicon size on the representative agarose gel of RT-PCR products of PRAB and PRB; product size: PRAB 327 bp, PRB 196 bp and β-actin 230 bp (Figure 1).

**Figure 2:**
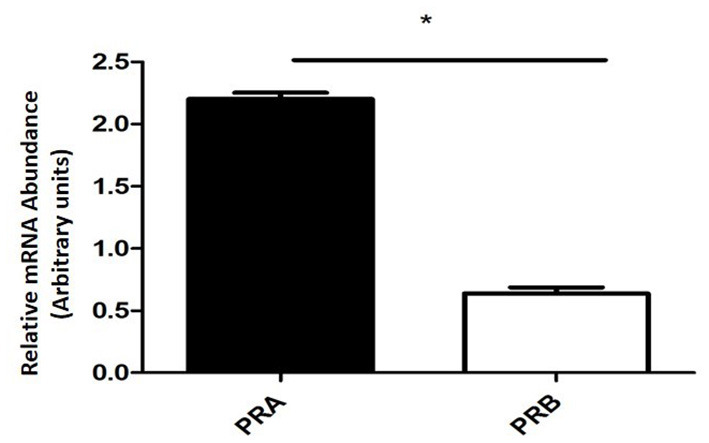
Relative mRNA abundance encoding PRA and PRB determined by qPCR in equine placental tissue samples (mean ± SEM, *n* = 6; *P < 0.0001).

The expression of PRA mRNA was significantly higher (*P* < 0.0001) than the PRB mRNA expression (Figure 2).

### PRA and PRB Protein Expression in Equine Placenta

**Figure 3:**
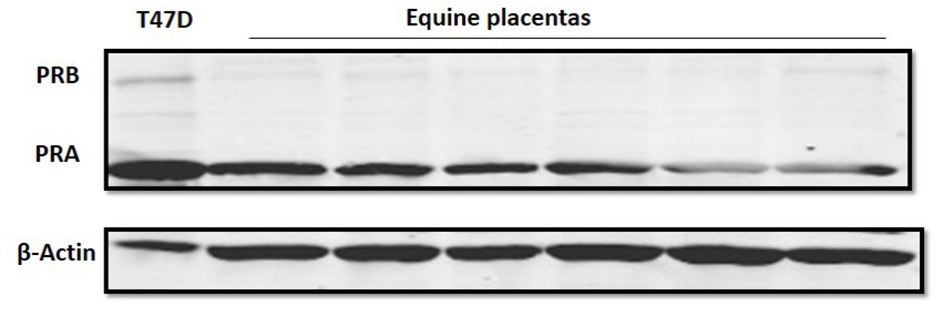
Progesterone receptor protein expression in equine placental tissue at term (*n* = 6). Immunoblot data show PRA and PRB, as well as β-actin as a loading control band. The T47D lysate was used as a positive control for PRA and PRB bands.

The Western blot of samples from equine placental tissue showed that the band intensity of PRA was markedly higher than that of PRB (Figure 3). The PRA and PRB bands were confirmed by the molecular weight and T74D whole cell lysate as a positive control.

**Figure 4:**
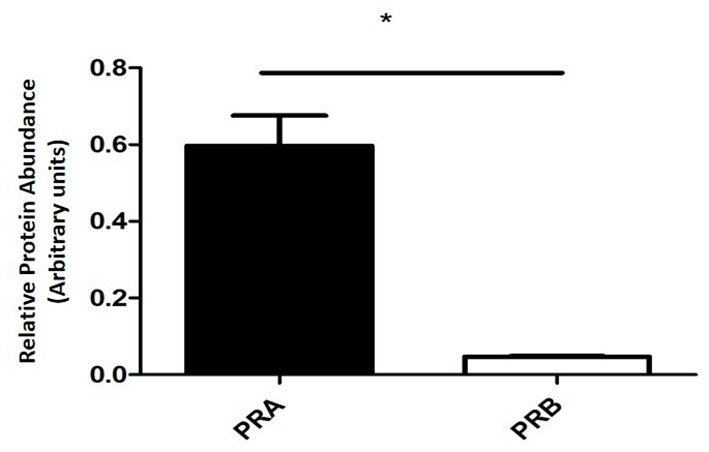
PRA and PRB protein relative abundance of equine placental tissue samples at term (mean ± SEM, *n* = 6; normalized to β-actin; *P < 0.0001).

In the equine placental tissue, the protein expression of PRA was significantly higher (*P* < 0.0001) compared to that of PRB (Figure 4).

## Discussion

To our best knowledge, this is the first study that determined both nPR isoforms; PRA and PRB transcripts and protein expression in the equine placenta at the normal term labor. In the present study, we demonstrated that the placenta expressed both nPR isoforms. The PRA was the dominant isoform expressed in the equine placenta, while PRB was hardly expressed or absent. This extremely low expression of PRB in equine placenta could be related to the initiation of parturition. These findings suggested that the placenta might be involved in the initiation of the parturition signaling. Moreover, the foaling process could be associated with the predominance of PRA, the inactive form of progesterone receptors over PRB expression, the active form of the receptors (progestin functional withdrawal), leading to stimulation of the inflammatory cascade and uterine contraction genes within the myometrial tissue.

Progesterone (P4) is the principal steroid hormone for pregnancy maintenance in most species. The protective action of P4 on pregnancy is mediated through nPRs in the target tissues. There are two isoforms of nPRs; both PRA and PRB are from the same gene, but transcribed by different promoters. PRA inactivates or suppresses, while PRB actively mediates the P4 actions (32). In the present study, both RT-PCR and Western Blot data revealed that the mRNA and protein expressions of PRA were profoundly higher than those of PRB in equine placenta at the normal term labor. These results are consistent with the findings of previous studies in human placenta (32, 33, 36). These investigators detected both nPR isoforms in the human placenta and found an increase in the expression of PRA compared to PRB expression following parturition. These results support the crucial role of the PRA in functional progesterone withdrawal and initiation of parturition. In pregnant women, PRA plays an important role in the parturition process and expulsion of the placenta via its direct and indirect actions on the inflammatory cascade and myometrial activation (37). PRA suppresses the anti-inflammatory actions of P4 mediated by PRB. Furthermore, PRA directly upregulates the pro-inflammatory gene expression (37). On the other hand, the findings of the present study were not consistent with the earlier findings in the human placenta that PRs did not exist in the term placenta (38, 39). This inconsistency may be due to the difference in the sensitivity of the methods and techniques used. These investigators used the nuclear exchange assay, which has difficulties in the detection of PR in the placenta due to high endogenous P4 concentration (30). Moreover, our findings are parallel to those observed in human myometrium at parturition. They found strong evidence for the superiority of PRA over PRB expression (14, 37). However, little is known about the nPR isoform changes in the equine placenta during parturition. An earlier study used immunohistochemistry (IHC) for the detection of the nPRs in different stages of equine pregnancy between 110 and 309 days (40). These researchers did not detect nPRs in the placental tissues at the late pregnancy stage. However, they did not use specific antibodies for different nPR isoforms. Furthermore, RT-PCR and Western Blot, the techniques we used in the present study, are more sensitive and specific for the detection of PRs compared to IHC (41).

In the present study, we found a marked increase in the PRA mRNA and protein expression compared to PRB in the equine placenta following foaling. These results followed those of recent studies in equine myometrium. They revealed that the experimentally induced placentitis in late pregnant mares is associated with the decrease of the PRB expression compared to PRA expression in the myometrium (20, 21). This drop in the nPRs expression is followed by an increase in the inflammatory cytokines and contraction-associated proteins, initiating a strong contraction of the myometrium and expulsion of the fetus (42). These findings emphasize the important role of PRA in initiating the labor. The dominance of PRA over PRB expression could be involved in the fetomaternal signaling of the initiation of parturition.

The findings of this study suggested that the equine placenta is involved in the progestins-nPRs signaling at labor. These findings supported the evidence from previous observations in humans that the placenta is one of the target tissues for the progestin effect (3, 30, 31). Our novel hypothetical scenario in foaling is that the parturition signal begins from the fetus, affecting the nPRs levels toward the PRA dominance within the placenta. That signal might be extended and activate the myometrium. The gestational and parturient physiology between human and equine are similar (19). This would support our hypothesis that the foaling is associated with the predominance of PRA over PRB expression (progestin functional withdrawal) which inhibits the progestins' protective function during pregnancy.

In conclusion, the present study showed that PRA, the less active or inactive form of nPRs, dominated over PRB, the active form of the receptors, in the mare placenta at term. The present findings raise the possibility that the PRA plays an important role in the parturition process in the mares including the expulsion of the placenta. Moreover, the equine parturition might be associated with a local functional progestin withdrawal in the placental tissues initiating the parturition process. These findings will help us understand the mechanisms by which progestins target the nPR isoforms. Moreover, they could help us understand the changes occurring in the term and preterm labor.

## Data Availability Statement

The original contributions presented in the study are included in the article/supplementary material, further inquiries can be directed to the corresponding author/s.

## Ethics Statement

The animal study was reviewed and approved by Institutional Animal Care and Use Committee of Iowa State University.

## Author's Note

The present study was performed at Iowa State University where AN was a visiting scholar.

## Author Contributions

WH, AN, and SS designed the study. SS collected and prepared the tissue samples. AN performed the mRNA and protein studies. AA performed the statistical analysis. AN and AH prepared and edited the manuscript. WH edited the final version of the manuscript. All authors read and approved the final version of the manuscript.

## Conflict of Interest

The authors declare that the research was conducted in the absence of any commercial or financial relationships that could be construed as a potential conflict of interest.
